# Multimodal Data Collection System for Driver Emotion Recognition Based on Self-Reporting in Real-World Driving

**DOI:** 10.3390/s22124402

**Published:** 2022-06-10

**Authors:** Geesung Oh, Euiseok Jeong, Rak Chul Kim, Ji Hyun Yang, Sungwook Hwang, Sangho Lee, Sejoon Lim

**Affiliations:** 1 Graduate School of Automotive Engineering, Kookmin University, Seoul 02707, Korea; gsethan17@kookmin.ac.kr (G.O.); euiseok_jeong@kookmin.ac.kr (E.J.); valance95@kookmin.ac.kr (R.C.K.); 2 Department of Automobile and IT Convergence, Kookmin University, Seoul 02707, Korea; yangjh@kookmin.ac.kr; 3 Chassis System Control Research Lab, Hyundai Motor Group, Hwaseong 18280, Korea; gazz@hyundai.com (S.H.); imprince@hyundai.com (S.L.)

**Keywords:** driver emotion recognition, multimodal, self-report, real-world driving

## Abstract

As vehicles provide various services to drivers, research on driver emotion recognition has been expanding. However, current driver emotion datasets are limited by inconsistencies in collected data and inferred emotional state annotations by others. To overcome this limitation, we propose a data collection system that collects multimodal datasets during real-world driving. The proposed system includes a self-reportable HMI application into which a driver directly inputs their current emotion state. Data collection was completed without any accidents for over 122 h of real-world driving using the system, which also considers the minimization of behavioral and cognitive disturbances. To demonstrate the validity of our collected dataset, we also provide case studies for statistical analysis, driver face detection, and personalized driver emotion recognition. The proposed data collection system enables the construction of reliable large-scale datasets on real-world driving and facilitates research on driver emotion recognition. The proposed system is avaliable on GitHub.

## 1. Introduction

In recent decades, the use of data-driven state-of-the-art techniques such as deep learning has increased interest in and performance of human affect recognition [1]. This has increased interest in the development of driver emotion recognition systems. Since driving is significantly affected by the driver’s emotions [2,3,4], driver emotion recognition studies have been conducted for various purposes such as driving safety, adjusting vehicle dynamics, and emotion elicitation of drivers [4,5,6]. All studies are affected by the quality and quantity of data. Therefore, research on quantitative and qualitative datasets for driver emotion recognition is being actively conducted [7,8,9,10,11,12,13,14].

Although large-scale and high-quality datasets are collected through various studies, the collection conditions vary significantly. First, the experimental environment is largely divided into simulation and real-world driving. Second, the modalities of collected signals are also diverse. When broadly classified, there are video, audio, bio-physiological, and controller area network (CAN) data. In detail, the position of cameras and microphones differ, and the collection list of biophysiological or CAN data is not unified. Lastly, the annotation of emotional states is various, which is critical for emotion recognition. The simplest way to classify a driver’s emotional state is by driving experiments (e.g., assume that heavy traffic on the urban is high stress, and light traffic on the highway is low stress) [7,8,9]. There is also an approach in which external annotators judge a driver’s emotional state based on the collected information about the driver. However, this approach has limitations in that it has a high-cost and requires others to report their emotional states [10,11]. In the self-reporting approach, drivers report their emotional states, but this should not interfere with the main task of driving. Hence, it is restricted to experiments through simulation or they have to report their emotional states after the completion of the experiments [12,13,14]. As previously stated, since data collection environments, measured data types, and annotation methods very, Zepf et al. have argued that a consistent dataset is needed to facilitate research on driver emotion recognition [15].

**Figure 1:**
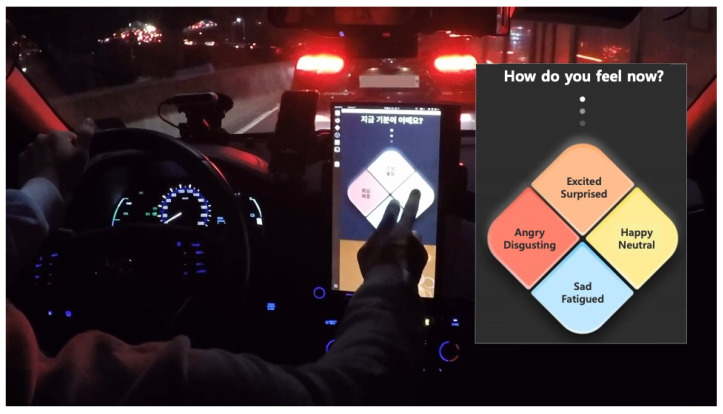
A scene in which a driver’s emotional state data is being collected during real-world driving using the proposed data collection system. The driver is self-reporting their emotional state by touching the HMI application mounted on the vehicle center fascia. The screenshot on the right is the English translation of the GUI of the HMI application implemented in Korean.

In this paper, we propose a data collection system that can be used for a variety of driver emotion recognition studies. The proposed system collects multimodal datasets such as videos from various views, audio, biophysiological, CAN data, and drivers’ emotional states, which are data representatively used for driver emotion recognition. A driver’s emotional state is collected by a driver self-reporting their emotional state while driving through a human–machine interaction (HMI) application. To realize a universal dataset, the collection experiment should be conducted in the real world environment, not through a simulator. To conduct a real-world driving experiment, it is necessary to prevent the behavioral and cognitive disturbances of drivers in advance to avoid potential traffic accidents. To prevent behavioral disturbance, the proposed system collects biophysiological data using wearable sensors, instead of biometric sensors attached to the body. The self-reporting application for minimizing cognitive disturbances comprises a haptic, acoustic response, and graphical user interface (GUI) based on user experience (UX). In addition, there are concerns about the reflection of strong bias during self-reporting due to false memories or the desire to impress others [15]. To address these concerns, we focused on making the self-reporting interaction occur periodically. All considerations for reliable data are detailed in Section 3. The data collection system is installed on a vehicle, and data collection is performed under real-world driving conditions. Figure 1 shows the data collection vehicle driving during real-world driving.

According to the real-world data collection experiment results using the proposed system, the experiment was completed without any accidents over four months. A large-scale dataset of over 122 h, 4446 km, and 787GB was collected, along with 6356 self-reporting data points of drivers while driving. Through the statistical analysis of the collected data, the imbalance of self-reported emotion labels and the need for personalized driver emotion recognition were confirmed. In addition, case studies of driver face detection and personalized single and multimodel driver emotion recognitions are presented, and comprehensive understanding is provided.

Our main contributions can be described as follows:

- We proposed a data collection system that can collect the multimodal data of drivers during real-world driving tasks. The proposed system is capable of collecting real-world driving big data for driver emotion recognition while considering the minimization of behavioral disturbances.
- The proposed system comprises an HMI application through which drivers can report their emotional states. This application is designed to collect selected emotional states from the driver without cognitive disturbance during real-world driving by utilizing the haptic, acoustic response, and GUI, and eliminating the bias problem that may occur with the self-reporting by setting the interaction period.
- We deployed the proposed system on a vehicle and collected high-quality multimodal sensor data without any accidents during real-world driving experiments for over 122 h. To demonstrate the validity of our collected dataset, we provided various case studies such as statistical analysis, driver face detection, and personalized single and multimodal driver emotion recognition.

The rest of this paper is organized as follows. Section 2 introduces related works on the data collection system for driver emotion recognition. Section 3 discusses the proposed data collection system in real-world driving. Section 4 provides data collection experiments, analysis of collected data, and case studies using the collected data. Section 5 concludes this work and describes further work. Appendix A describes details of terminologies and variables used in this paper.

## 2. Related Works

Driver state recognition research is being conducted from various viewpoints, from the recognition of inattention [16], distraction [17], stress [5], and behavior [18] for safety to readiness [19] for autonomous driving. This has resulted in research on driver emotion recognition, along with the improvement of data-based human emotion recognition performance [20,21,22]. Data used for driver emotion recognition is classified into video [11], audio [10], biophysiological [12], and CAN data [15]. In most cases, these data are not used alone but are fused to recognize driver’s emotional states [6,7,8,9]. However, real-world driving data resources that account for data types do not exist. Ma et al. [11] only collected the video of a driver’s face, and CIAIR [23] and DriveDB [7] collected video, audio, and biophysiological data, excluding CAN data. UTDrive DB collected various CAN data, along with video and audio but did not collect bio-physiological data [8]. In this study, we propose the various multimodal data collection system in real-world driving.

Emotion annotation data are as important as sensor data in driver emotion recognition. To annotate a driver’s emotional state, three major methods are employed: experimental context, external annotators, and self-reports. The experimental context is the simplest way to annotate an emotional state by estimating the driver’s emotional state with the driving situation or environment, e.g., annotate the driver’s stress level by road type or congestion level [7,8,9]. Since this approach presupposes strong assumptions, there are limitations in annotating an accurate emotional state. Although using external annotators requires additional manpower and cost, it enables objective annotation. Jones and Jonsson recorded a driver’s speech while driving using a simulator, and an external annotator annotated the driver’s emotional state by listening to the recorded speech for driver emotion recognition [10]. Ma et al. developed an annotation tool to allow external annotators to annotate two emotion categories at five levels each based on driver face images collected during real-world driving [11]. This approach also has limitations in that experienced and trained annotators are required. Because self-reporting is an approach to self-report how drivers feel while driving, it can overcome the limitations of other approaches. However, driving is a task that requires considerable concentration, and drivers’ self-reporting while driving affects the experiment. Hence, most self-reporting is performed immediately after the driving experiments. Taib et al. [13] and Ihme et al. [14] conducted a driving simulation experiment for driver frustration and asked participants who drove for self-reporting information after the experiment. Taib et al. used a 9-point Likert scale and Ihme et al. used a self-assessment manikin (SAM) [24] for self-reporting. Kato et al. proposed a self-report application that can visualize data and selected the driver’s emotional state while driving [12]. The proposed application enables a driver’s self-reporting to be performed in real time while driving, not after the experiment. This application was only used in a simulation experiment, and to use it in real-world driving experiments, additional safety considerations are required. In addition, concerns about subjective biases that may be included in self-reports are another challenge to overcome [15]. In this study, we propose an HMI application that allows drivers to safely report their emotional states while real-world driving.

## 3. Proposed Work

**Figure 2:**
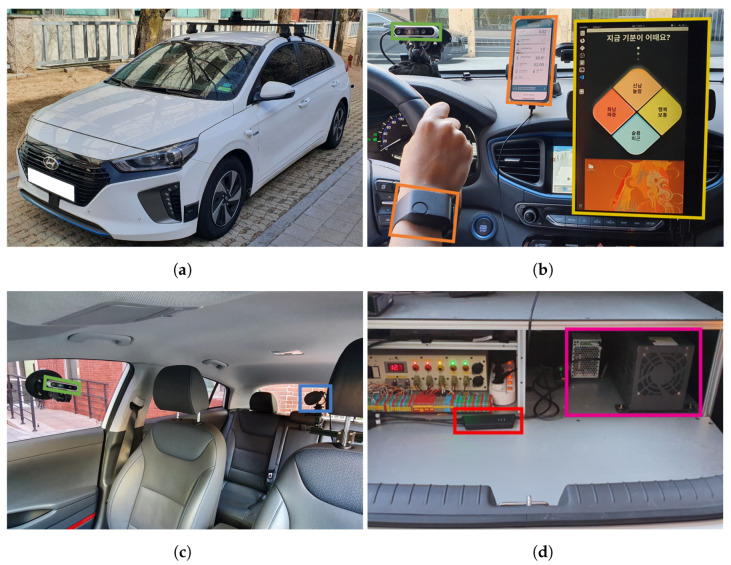
Figures of the dataset collection system hardware interface build in the vehicle. (**a**) Vehicle exterior; (**b**) Inside view of the vehicle center fascia; (**c**) Inside view of the vehicle passenger seat; (**d**) Vehicle trunk. Two cameras are installed to collect the image data of a driver’s face and posture (green). A microphone is installed on the right side of the driver seat’s headrest to collect audio data in the cabin (blue). Wristband-type wearable sensor is worn on the driver’s wrist to collect the driver’s bio-physiological data, and the collecting status can be monitored through a smartphone (orange). The CAN interface device supports the collection of vehicle CAN data (red). The monitor installed on the center fascia is a touch screen for interaction with the driver (yellow). The computer installed in the trunk of the vehicle integrates the collected data (magenta).

**Figure 3:**
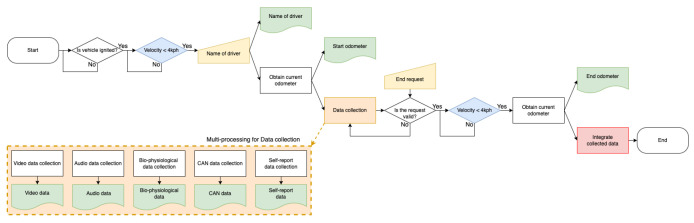
Flow chart of the proposed data collection system during real-world driving.

In this section, a system that enables the simultaneous collection of videos, audio, biophysiology, and CAN data during real-world driving is described. The system also includes an HMI application that interacts with the driver and collects the driver’s emotional state. In other words, this section demonstrates methods for developing hardware and software systems for a multimodal dataset based on self-reporting in real-world driving for driver emotion recognition. All systems are built into the vehicle, as the data collection is performed under driving conditions. We used an IONIQ 1.6 Hybrid vehicle (Hyundai, Seoul, KR, https://www.hyundai.com/, accessed on 31 March 2022) shown in Figure 2a as the base environment for building the proposed system. Figure 3 shows the flowchart of the entire system. When the system starts, the first thing to check is whether the vehicle is ignited. The system is designed to start after the vehicle is ignited because the surge voltage generated when the vehicle is ignited can reduce the quality of data collected using electronic sensors. In addition, for safety reasons, whether the vehicle is stopped before starting and ending the system is checked (blue rhombus in Figure 3). This prevents the driver from operating the system while driving. After confirming that data collection is possible, two types of metadata are requested before the main data collection. One is the name of the driver, which must be input by the driver manually. The other is the current odometer, which can be obtained automatically via vehicle CAN data. After obtaining the current odometer and treating it as the starting odometer, the main data collection process starts. The main data collection process uses multiprocessing to efficiently collect different multimodel data (orange rectangle in Figure 3). When a suitable end request is input into the system by the driver, the main data collection process is terminated, and if the vehicle is stopped, the vehicle odometer is obtained once more and treated as the ending odometer. Finally, all data, metadata, and collected data (green box in Figure 3) are integrated into one dataset (red rectangle in Figure 3), and the entire system is shut down. All processes in the proposed system are performed using a computer, shown as Figure 2d. The proposed system is released as an open source repository on GitHub (https://github.com/KMUIMLAB/DMS, accessed on 27 May 2022) and the details of each data type for multimodal data collection are discussed in the following sections.

### 3.1. Video

We use two RealSense D435i cameras (Intel, Santa Clara, CA, USA, https://www.intel.com/, accessed on 31 March 2022) to collect video data composed of various modalities. The RealSense camera provides a maximum of three video modalities: red, green, and blue (RGB), infrared (IR), and depth. In addition to the RGB image, the IR image, which is robust to environment changes, such as illumination changes, is essential in real-world driving. One camera is installed on the dashboard to capture the driver’s face, as shown in Figure 2b, and the other is installed on the top of the passenger seat window to capture the driver’s posture, as shown in Figure 2c. Since the sample rate of the camera can be set, we set it as $R_{v}$ Hz. Alternatively, each camera sequentially captures $R_{v}$ individual images per second.

### 3.2. Audio

The CVM-VM10 II microphone (CoMica Technology, Shenzhen, Guangdong, CN, https://www.comica-audio.com/, accessed on 31 March 2022) was used to collect audio information in the cabin while driving. To collect data with audio information similar to what the driver hears, the cardioid condenser microphone was selected and placed close to the driver’s ear. To minimize noise and vibrations that occur during real-world driving, the microphone was installed on the right side of the driver’s seat headrest, along with the shock mount and wind muff, as shown in Figure 2c. The audio data collection system collects $R_{a}$ audio data samples per second until the system stops according to the sample rate, $R_{a}$ Hz.

### 3.3. Biophysiological

To collect biophysiological data of the driver, the biometric sensor must be in contact with the driver’s body. The attached sensor may cause behavioral disturbances, resulting in potential accidents. For safe biophysiological data collection during real-world driving, it is necessary to prevent behavioral disturbances in advance, and we used an E4 wristband (Empatica, Boston, MA, USA, https://www.empatica.com/, accessed on 31 March 2022), as a solution. The E4 wristband (E4) is a wearable biometric sensor and is used as an alternative sensor while exhibiting similar data quality 85% of the time compared to the clinician standard device [25]. As a result of comparing the E4 and laboratory biometric sensor data in terms of emotion recognition performance, similar accuracy was realized [26]. Hence, we used the E4 for biophysiological data collection during real-world driving. E4 provides skin temperature, electrodermal activity (EDA), photoplethysmography (PPG), and 3-axis acceleration of the band, along with interbeat interval (IBI) and heart rate (HR) through postprocessing. As shown in Figure 2b, biophysiological data collection is possible by simply wearing E4 on the wrist while driving, and real-time monitoring is also possible using a mobile device through the application provided by E4. Unlike video or audio data, E4 collects each data at an optimized sampling rate, so no separate setting is required. Each sample rate is shown in Table 1.

### 3.4. CAN

The method of mounting additional sensors or collecting on-board diagnostics (OBD) signals can also be used to access vehicle signals; however, since we can access vehicle CAN, we can collect vehicle signals with the CAN interface device. CAN is a message-based protocol designed to allow vehicle controllers to communicate with each other. The USBcan Pro 2xHS v2 (KVASER, Mission Viejo, CA, USA, https://www.kvaser.com/, accessed on 31 March 2022) is a CAN interface device used to access vehicle CAN signals to collect vehicle data. As shown in Figure 2d, the device is located in the trunk of the vehicle and connects the vehicle CAN line to the computer. Among the many signals on CAN, we select key signals closely related to the driver. Since the selected key signals are updated according to the set cycle time, the sample rate of CAN data, $R_{c}$, is set according to the cycle time. The collected key data and the sample rate are presented in Table 1.

### 3.5. HMI

Drivers’ emotion annotation is essential in datasets for driver’s emotion recognition. Although external annotators or the experimental context can be employed to estimate and annotate drivers’ emotional states, we focused on annotating the driver’s emotional state using reports from the driver rather than via estimation. This method is called self-report and will be performed in real-world driving experiments. It must be designed with an emphasis on safety. Requiring drivers to report driving conditions may cause cognitive disturbances, probably leading to severe traffic accidents on the road.

To minimize cognitive disturbances, we proposed the HMI application that periodically interacts with the driver through haptic and acoustic response and receives the emotional state response from the driver. We used a TFX133T DEX monitor (HANSUNG, Seoul, KR, https://www.monsterlabs.co.kr/, accessed on 31 March 2022), and the touch screen has a built-in speaker to realize haptic and acoustic responses. The screen was installed on the center fascia of the vehicle, as shown in Figure 2b. When data collection starts, the HMI application requests that the driver report their emotional state with a sound announcement as follows: “Please enter your current state”. If there is no response from the driver for $I_{rr}$ seconds from the request, the application requests once more with the same sound announcement. If there is no response from the driver within $I_{s}$ seconds from the first request, not to disturb the driver, it is treated as a nonresponse with a sound announcement as follows: “The input is delayed, so it enters in a nonresponse state”. This skipping process is essential as frequent response requests can interfere with safe driving. The driver can input an answer by only touching the screen, and when the input is completed, the input emotional state is displayed on the screen in large fonts; and at the same time, a sound announcement is provided as follows: “Your input is complete”. This feedback minimizes confusion for the driver.

In addition to cognitive disturbances, self-reported emotion labels have limitations in that they reflect strong bias because of false memories or the desire to impress other people [15]. Repeated sampling in real-time is necessary to minimize this bias [27]. That is, the self-reporting requests should be continuously made at periodic intervals. Hence, the proposed HMI application continuously requests the response at an interval, $I_{r}$, from when driving starts to when it ends. The interval between response requests, $I_{r}$, is tuned through test driving. Moreover, our system allows the driver to report their emotional states at any time by touching the screen even between response intervals. This feature enables logging drivers’ rapidly changing emotional changes in real-world varying driving conditions.

The proposed HMI application can apply any representative emotional states as long as they are discretely expressed states. However, since the driver has to choose the most similar to their current emotional state among them, cognitive disturbances can occur if there is difficulty in choosing an emotion no matter how well the interaction with the driver is completed. Therefore, the discrete representative emotional states should be simple, not numerous, and suitable for the driving situation.

### 3.6. GUI

We propose a GUI design to reduce drivers’ cognitive disturbance in self-reporting through HMI while driving. To propose UX-based GUI of the HMI application, the following four representative driver emotional states by referring to the emotions that can be induced in a driving situation [28] are suggested.

- Happy|Neutral;
- Excited|Surprised;
- Angry|Disgusting;
- Sad|Fatigued.

**Figure 4:**
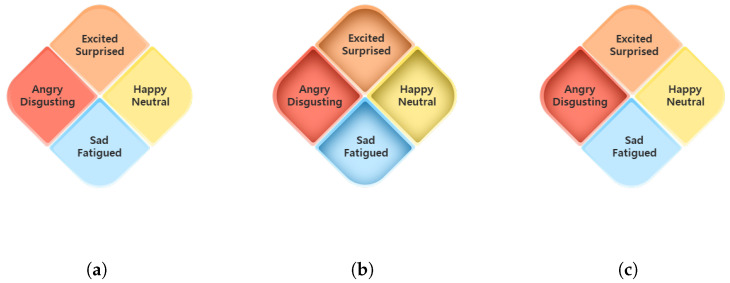
GUI of HMI application for self-reporting of driver emotional state. (**a**) GUI in default; (**b**) GUI in touch; (**c**) GUI example where “Angry|Disgusting” state is touched.

The proposed GUI designs are shown in Figure 4. There are two factors to consider in the GUI design: the layout and color of the emotional states. The layout of the emotional states refers to the valence–arousal plane, a popular concept used in emotional representation [29]. Based on the division of the x-axis into pleasure and misery in the valence–arousal plane, we placed “Happy|Neutral” and “Angry|Disgusting” on the right and left of the screen: “Happy|Neutral” is on the right and “Angry|Disgusting” is on the left. Based on the division of the y-axis into arousal and sleepiness in the valence–arousal plane, we placed “Excited|Surprised” and “Sad|Fatigued” on the top and bottom of the screen: “Excited|Surprised” is on the top and “Sad|Fatigued” is on the bottom. The overall layout of the emotional states is in the form of a rhombus, as shown in Figure 4. In the GUI shown in Figure 4, each emotional state is expressed in different colors. The correlation between basic colors and human psychological state was identified, and states that can be felt by humans were classified according to color characteristics [30]. Based on this, appropriate colors were used for each emotional state. The GUI design provides not only a default GUI, as shown in Figure 4a, but also a touch GUI, as shown in Figure 4b. Therefore, when the driver inputs the current emotional state by touching the screen, it provides visual feedback, as shown in Figure 4c, along with the sound announcement. The UX-based GUI of the HMI application gives the driver more accurate intuition about the proposed representative emotional states.

## 4. Experiments

This section presents the details of the data collection experiment conducted on the basis of the proposed data collection system and some case studies based on the collected data from the experiment.

### 4.1. Data Collection Experiment

Motivated by the need for a dataset in real-world driving, the data collection experiment with the proposed system described in Section 3 was conducted on the road. During real-world driving, the cameras are used to capture RGB and IR modalities at the sample rate, $R_{v}$, of 15 Hz, and audio data are collected at the sample rate, $R_{a}$, of 44,100 Hz. Biophysiological data are collected, as described in Section 3.3. The following CAN data signals are collected: accelerator pedal position, brake pedal position, steering wheel angle, yaw rate, longitudinal acceleration, and lateral acceleration. All CAN data are collected at the sample rate, $R_{c}$, of 100 Hz. The self-reportable application collected the driver’s emotional state in five states involving four representative emotional states mentioned in Section 3.5 and nonresponse. The response request time interval, $I_{r}$, is set to 60 s, and then the sample rate of self-reported emotion label, $R_{s}$, is $\frac{1}{60}$ Hz. Because the driver is encouraged to self-report whenever there is a change in their emotional state even without that response request, the self-reported emotional state annotation includes information on the driver’s emotional change for unexpected or urgent events. The rerequest time interval, $I_{rr}$, and the skip time interval, $I_{s}$, are set to 10 and 20 s, respectively. All interval times have been adjusted through several test drives in real-world driving, so that there is no safely issue. Details, including save format and unit for all data collected through the experiment, are described in Table 1.

**Table 1:** Details of data collected by experiment.

Data		Sample Rate (Hz)	Format	Unit
Video	RGB-front	15	.avi	-
	RGB-side	15	.avi	-
	IR-front	15	.avi	-
	IR-side	15	.avi	-
Audio	-	44,100	.wav	-
Bio-physiological	Skin temperature	4	.csv	${}^{\circ }C$
	EDA	4	.csv	μS
	PPG	64	.csv	nW
	IBI	-	.csv	s
	HR	1	.csv	bpm
	3-axis acceleration	32	.csv	$\frac{1}{64}g$
CAN	Accelerator pedal position	100	.csv	%
	Brake pedal position	100	.csv	%
	Steering wheel angle	100	.csv	${}^{\circ }$
	Yaw rate	100	.csv	rad/s
	Longitudinal acceleration	100	.csv	$m/s^{2}$
	Lateral acceleration	100	.csv	$m/s^{2}$
Self-reported emotions	Emotional state	no less than $\frac{1}{60}$	.csv	-

To address the lack of long-term datasets, the experiment was conducted with a few people who could participate continuously for a long period. Four males participated in the experiment for four months from July 2021 to October 2021. The detailed information of these participants is described in Table 2.

During these four months, a large-scale dataset was collected by the participants’ driving in wild, uncontrolled conditions. The weather conditions were divided into four categories, and the proportions are as follows: Sunny: 20.4%, Cloudy: 40.6%, Overcast: 11.8%, Rainy: 27.3%. Because safety is considered in the proposed data collection system, no accidents occurred during this period, and according to the data collection experiment results, the total experiment time was 122 h 15 min, the total driving mileage was 4446 km, the total number of self-reported emotion labels was 6356, and 787 GB data were collected.

### 4.2. Case Studies

This section presents some case studies using the collected multimodal dataset for driver emotion recognition. Section 4.2.1 discusses the detailed analysis of the dataset collected in real-world driving. Section 4.2.2 and Section 4.2.3 present case studies of driver emotion recognition using single-modal or multimodal inputs.

#### 4.2.1. Statistical Analysis

**Figure 5:**
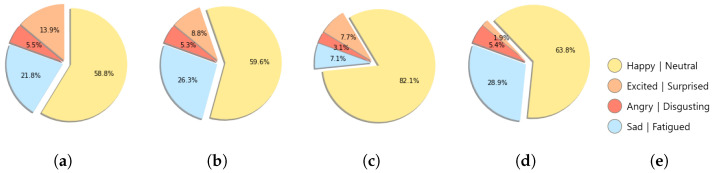
Pie charts for self-reported emotion label proportion by driver. (**a**) Driver A; (**b**) Driver B; (**c**) Driver C; (**d**) Driver D; (**e**) Legend of the pie charts.

In this section, we discuss the detailed analysis results for the collected dataset in the real-world driving experiment. Figure 5 depicts the self-report proportion for each driver as a pie chart. The emotion with the highest proportion was “Happy|Neutral”. More than 50% of the drivers’ self-reported emotion labels are “Happy|Neutral”, and they often account for up to approximately 82%. The proportion of the other three emotions varies by the driver, but it accounts for a small proportion compared to the “Happy|Neutral”.

**Figure 6:**
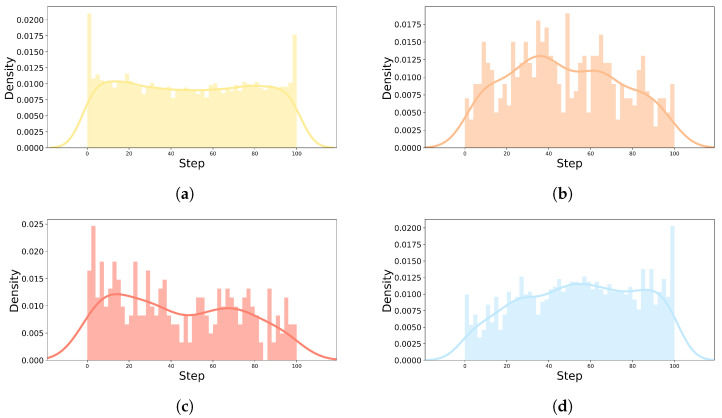
Distribution of self-reported emotion labels in real-world driving. (**a**) Happy|Neutral; (**b**) Excited|Surprised; (**c**) Angry|Disgusting; (**d**) Sad|Fatigued.

**Figure 7:**
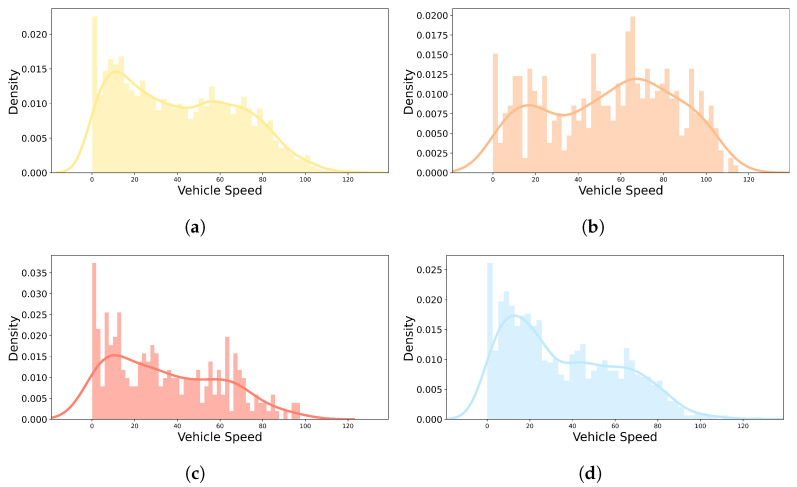
Distribution of vehicle speed by self-reported emotion labels in real-world driving. (**a**) Happy|Neutral; (**b**) Excited|Surprised; (**c**) Angry|Disgusting; (**d**) Sad|Fatigued.

To confirm the self-reported emotion label tendency of each emotion, the distribution of self-reports and vehicle speed by emotion for all drivers is depicted in Figure 6 and Figure 7. In Figure 6, the start and end of all individuals driving were normalized from 0 to 100 steps and divided into 50 sections. The number of self-reported emotion labels for each section is displayed as a histogram and kernel density estimate plot to evaluate the distribution by emotion. “Happy|Neutral” had several distributions at the start and end of the driving, and had an even distribution throughout the driving process, as shown in Figure 6a. Overall, “Excited|Surprised” and “Angry|Disgusting” had an irregular distribution. “Excited|Surprised” seemed to have a greater variance than “Angry|Disgusting”, as shown in Figure 6b,c, and it is judged that “Excited|Surprised” was more maintained when the emotion was induced than “Angry|Disgusting”. As shown in Figure 6d, the distribution of “Sad|Fatigued” emotion increases toward the middle and late stages of driving. Figure 7 shows the number of self-reported emotion labels at that vehicle speed with a histogram and kernel density estimate plot to evaluate the distribution of vehicle speed by self-reported emotion labels. “Happy|Neutral” had high distributions from 0 to about 15 kph, and an even distribution throughout the driving process, as shown in Figure 7a. In Figure 7b,c, the fact that the vehicle speed had a relatively irregular distribution compared to “Happy|Neutral” and “Sad|Fatigued” in “Excited|Surprised” and “Angry|Disgusting” is a common feature with the distribution of self-reported emotion labels in Figure 6. As shown in Figure 7d, the distribution of the “Sad|Fatigued” emotion had a particularly high distribution from 0 to about 30 kph. Based on the distribution of self-reports and vehicle speed by emotion (especially in Figure 6a), “Happy|Neutral” was the default emotion and the others were induced while driving.

In addition to self-reported emotion label data, we used the statistical hypothesis test to analyze the significance of the collected sensor data. We built the null hypothesis ($H_{0}$) that the structured data collected did not differ according to the self-reported emotion label and confirmed the difference by the emotion of each structured data through a Kruskal–Wallis H test [31,32]. According to the Kruskal–Wallis H test results, if the significance probability expressed as the *p*-value is less than the significance level, 0.05, the null hypothesis ($H_{0}$) can be rejected and the alternative hypothesis ($H_{1}$) can be accepted as true. The statistical significance by self-reported emotion label of each data is described using the *p*-value and which hypothesis was accepted as true in Table 3. If the statistical significance between the four self-reported emotion labels is confirmed by the Kruskal–Wallis H test, it is also necessary to confirm how many of the pairs show statistical significance through the post-hoc test. We confirmed the statistical significance of a total of six self-reported emotion label pairs through the Mann–Whitney U test [33,34], a nonparametric statistical hypothesis test, and the total number of the null hypothesis ($H_{0}$) rejection pairs is also listed in Table 3. As shown in Table 3, all collected structured data had statistically different distributions for self-reported emotion labels, and three or more pairs out of six pairs were statistically significant.

Although the statistical hypothesis test results can explain the significance of the emotion recognition of the collected sensor data, another aspect that requires analysis is whether there is a significant distribution difference according to the driver. Therefore, the same statistical hypothesis test as above was repeated by separating the data for each driver, and the results are shown in Table 4. EDA and steering wheel angle are the only structured data with the same results for all drivers. Not only were the post-hoc results different, but also the results of determining whether to reject the null hypothesis were different for each driver. That means the collected data significantly vary from driver to driver. This may be because each driver has a different way of expressing their emotions while driving. Therefore, different data will be required to recognize each driver’s emotion. In other words, emotion recognition research requires personalization.

#### 4.2.2. Driver Face Detection

**Figure 8:**
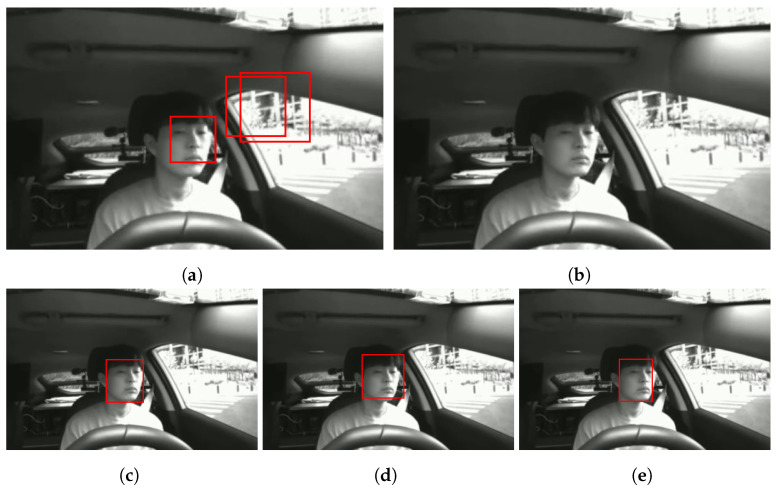
Example of the detection results of five face detectors. The bounding boxes (red) are face detection results. (**a**) Haar; (**b**) Dlib; (**c**) OpenCV; (**d**) MMOD; (**e**) MTCNN.

One of the most common approaches to recognizing a driver’s emotional state is using face images. Studies adopting this approach generally use a well-known face detector to crop only the face image from the driver’s frontal image and use it as input data. The most popular face detectors have proven their performance only on in-the-wild datasets such as FDDB [35] or WIDER FACE [36]. Thus, we evaluate the performance of five popular face detectors, Haar [37], Dlib [38], OpenCV [39], MMOD [40], and MTCNN [40], on detecting the driver’s front image in the collected real-world driving dataset. First, the detection results of the five detectors for the collected IR-front images were output and qualitatively compared. Figure 8 is an example of the detection results of the five detectors. According to the results, Haar has a high false positive rate, i.e., nonfaces are detected, and Dlib has a high false negative rate, i.e., faces are not detected. In contrast to Haar and Dlib, other detectors are capable of detecting the driver’s face to a similar degree.

**Figure 9:**
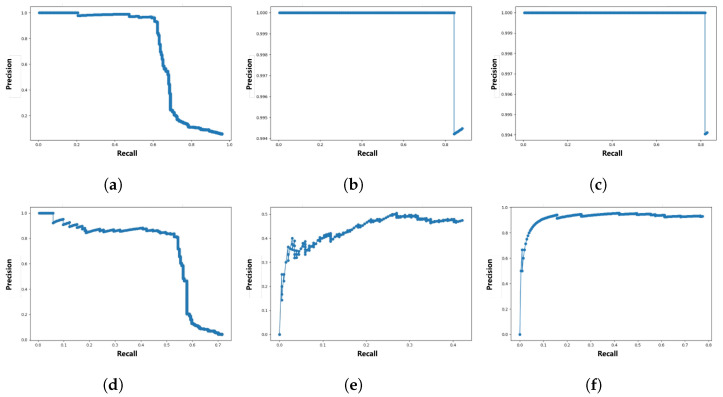
PR curve for face detectors capable of detecting the driver’s face. The thresholds are 0.5 and 0.75. (**a**) OpenCV, threshold is 0.5; (**b**) MMOD, threshold is 0.5; (**c**) MTCNN, threshold is 0.5; (**d**) OpenCV, threshold is 0.75; (**e**) MMOD, threshold is 0.75; (**f**) MTCNN, threshold is 0.75.

For accurate performance comparison of the similar three face detectors, we selected 200 different images and labeled face bounding boxes. If the intersection over union (IoU) value between the labeled bounding box and the detection bounding box is greater than or equal to the threshold, it is considered true positive (TP); if the IoU value is less than the threshold, it is considered false positive (FP). Figure 9 shows the precision–recall (PR) curve drawn using the considered TP and FP. Quantitative performance comparison of face detectors can be made with the average precision (AP) value calculated by the area under the PR curve. Depending on whether the threshold is 0.5, 0.75, or 0.95, AP performance is expressed as AP50, AP75, or AP95, respectively. Refer to Table 5 for detailed comparison results. Since the inference speed of the face detector is as important as detection accuracy, Table 5 describes the inference speed and the GPU specifications.

**Figure 10:**
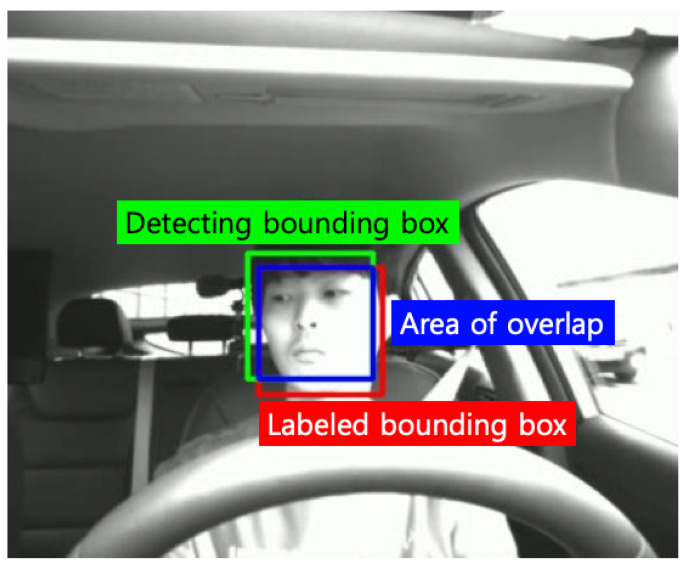
Example image with IoU of 0.68. Area of union (green and red) is 7441, and area of overlap (blue) is 5040.

OpenCV has the fastest inference speed, but its detection performance is low. For MMOD and MTCNN, AP50 is at a similar level, but at AP75, the detection performance of MMOD decreases rapidly. Although the AP75 performance of MTCNN is inferior to AP50, it is insignificant. Conversely, in the case of inference speed performance, MMOD significantly outperforms MTCNN. Since the inference speed of MTCNN is also insufficient, it seems appropriate to use a suitable face detector as the driver face detector depending on the purpose or computational sources. In terms of AP95, the performance of all detectors is 0.0. This is due to the small area occupied by the driver’s face in the driver’s front image, and the IoU value may not exceed the threshold value of 0.95 due to differences in determining whether only the eyes and nose are included, or including the forehead or chin when the bounding box is labeled. Figure 10 shows an example image of the detected and labeled driver face bounding boxes with an IoU value of 0.68, it detects the driver’s facial expression sufficiently. In face detection for driver emotion recognition, the threshold should not be as high as 0.5 or 0.95. Therefore, we crop the face image using the MMOD face detector, which achieved the highest detection performance in AP50 for driver emotion recognition, as discussed in Section 4.2.3.

#### 4.2.3. Personalized Driver Emotion Recognition

This section discusses the results of personalized driver emotion recognition utilizing single or multimodal data. Since individual driver data are required for personalized driver emotion recognition training, the data required to complete the training should be as small as possible, and the performance of the trained recognition model should be preserved for as long as possible. Therefore, the collected data are sorted in ascending order of mileage, and the mileage for completing the collection of training data, *K*, is determined. The data collected during *K* km driving from the initial mileage for each individual are used as training data, and the data from thereafter to the last data are used as test data. We set the completing mileage for the training data, *K*, to 500 km, and to obtain more test data than training data, we experimented with drivers A and B, who collected data over 1000 km.

**Figure 11:**
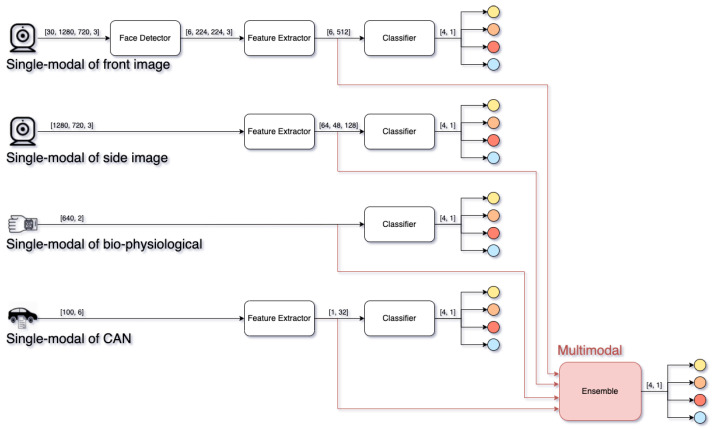
Deep learning-based personalized driver emotion recognition model.

We proposed a personalized driver emotion recognition model based on deep learning networks that recognize a driver’s emotional state using four multimodal inputs: front and side image, biophysiological, and CAN data. The proposed model is trained and verified using only individual data, and, as shown in Figure 11, each multimodal input performs single-modal emotion recognition and multimodal emotion recognition through an ensemble model. Each single-modal model and multimodal recognition model are described as follows.

- Single-modal of front image ($S_{f}$): The single-modal recognition model of the front image uses front IR images for 2 s from 4 s to 2 s before the driver’s self-reporting. Because RGB images are vulnerable to changes in illuminance, IR images that can always capture a stable image are used as input. From 2 s before self-reporting, it shows uniform motion for self-reporting, so it is excluded from the input data. The input images are evenly time-divided into six equal parts and input to a face detector; the MMOD-based face detector outputs one cropped face image with the highest confidence value for each input. The cropped images are resized to the input shape of the feature extractor and sequentially fed into a feature extractor and a classifier based on CAPNet [41]. Because the classification form is different from that of CAPNet, only the number of units in the top layer of the classifier is modified to the number of representative driver emotional states. The last activation function is softmax and outputs the probability of each representative driver emotional state.
- Single-modal of side image ($S_{s}$): The single-modal recognition model of the side image uses the side IR image captured 2 s before self-reporting. The reason for using the image from 2 s ago is the same as that for using the front image. The input image is fed into a feature extractor based on AlphaPose [42]. The feature extractor consists of layers up to just before outputting feature points in the form of histograms in AlphaPose. The classifier consists of a global max pooling layer and fully connected layers. The top layer of the classifier is the same as other classifiers to output the probability of each representative driver emotional states.
- Single-modal of biophysiological ($S_{b}$): The single-modal recognition model of biophysiological data uses the PPG and EDA data for 10 s before the driver’s self-reporting. Since PPG and EDA have different sample rates, up-sampling using linear interpolation is applied to the EDA data to match the input shape. The biophysiological input is directly fed into the classifier without a feature extractor to output the probability of each representative driver emotional state. The classifier is composed of the fully connected and batch normalization layers.
- Single-modal of CAN ($S_{c}$): The single-modal recognition model of CAN data uses all collected signals for 10 s before the driver’s self-reporting. The input data are down-sampled by a tenth before being fed into the feature extractor. The feature extractor is an encoder of long short-term memory-based autoencoder that extracts the feature vector for driving propensity. The classifier consists of fully connected layers and a dropout and outputs the probability of each representative driver emotional states by receiving the feature vector.
- Multimodal (*M*): The multimodal recognition model uses the input vectors of each classifier of single-modal as input vectors. The model is a deep learning-based ensemble model that outputs the probability of each representative driver emotional states by fusing all input vectors. The feature vectors of the front image, CAN, and side image are flattened using flatten and pooling layers. The flattened vectors are concatenated using the concatenate layer. The concatenated vector undergoes the normalization, fully connected layers, and softmax activation function to become the final output. The input modalities to fuse can be chosen, and the modals are denoted by a subscript, e.g., $M_{fb}$ is the ensemble model that fuses the front image and biophysiological data. We evaluated three or more input modal combinations for multimodal models.

It is necessary to define a loss function when training the proposed models. Because the self-reported emotion label has data imbalance, as described in Section 4.2.1, high performance cannot be expected if a typical loss function is used such as cross entropy. We overcome the data imbalance problem by making the precision and recall differentiable by computing the likelihood values of TP, FP, and false negative (FN) using probabilities. The loss function we used is shown as follows:

(1) $$L\left(y,\hat{y}\right)=1-\frac{1}{N}\left(\frac{p_{1}^{\mathrm{TP}}}{p_{1}^{\mathrm{TP}}+p_{1}^{\mathrm{FP}}+\epsilon }+\sum_{i=2}^{N}\frac{p_{i}^{\mathrm{TP}}}{p_{i}^{\mathrm{TP}}+p_{i}^{\mathrm{FN}}+\epsilon }\right)$$

(2) $$p^{\mathrm{TP}}=y\circ \hat{y}$$

(3) $$p^{\mathrm{FP}}=\left(\left[\begin{array}{c} 1.\\ 1.\\ 1.\\ 1. \end{array}\right]-y\right)\circ \hat{y}$$

(4) $$p^{\mathrm{FN}}=y\circ \left(\left[\begin{array}{c} 1.\\ 1.\\ 1.\\ 1. \end{array}\right]-\hat{y}\right)$$

where y and $\hat{y}$ represent a one-hot vector of the self-reported emotion and predicted emotion, respectively, where the first element of each vector represents the default emotion, “Happy|Neutral”. $p^{\mathrm{TP}}$, $p^{\mathrm{FP}}$, and $p^{\mathrm{FN}}$ are the likelihood values of TP, FP, and FN, respectively, where ∘ is an element-wise product.

Equation (1) is a loss function for increasing the precision of default emotion and for increasing the recall of induced emotions, where *N* represents the total number of representative emotions, and ϵ represents a very small value that prevents the precision or recall values from going to infinity. This loss function, $L(y,\hat{y})$, can be used for backpropagation by probabilistically expressing the precision and recall for each prediction class. It increases precision for the majority class, the default emotional state, and increases recall for minority class, inducible emotional states.

The evaluation results with test data are in terms of F1 score, precision, and recall, and are described for each driver. As mentioned in Section 4.2.1, since the representative driver emotional states are divided into default and inducible emotions, the recognition performance of inducible emotions is evaluated first. Table 6 and Table 7 summarize the performance of inducible emotion recognition between default and inducible emotions for each driver. The highest recognition performance is the F1 score 0.698 of $S_{s}$ for Driver A and 0.667 of $M_{sbc}$ for Driver B. As expected in Section 4.2.1, the input modals with the best performance for each driver differed. Driver A achieved the best performance in a single front image, and Driver B achieved the best in a side image, biophysiological, CAN data combination. However, their performance was similar. Driver B had similar performance between all evaluated models from 0.562 to 0.667. For Driver A, models without CAN data had a similar performance from 0.613 to 0.696, but models with CAN data such as $S_{c}$, $M_{fsc}$, $M_{fbc}$, $M_{sbc}$, and $M_{fsbc}$ had a significantly lower performance from 0.228 to 0.469. Driver B can interpret that when inducible emotions are induced while driving, emotions are expressed overall in the front and side images and biophysiological, and CAN data, whereas driver A can interpret that the induction of emotion is not expressed in CAN data. These results may support the fact that driver emotion recognition necessitates personalization.

The performance of driver emotion recognition among the inducible emotions for each driver is also summarized. The recognition performance for each of the three inducible emotions and the average of three F1 scores are described in Table 8 and Table 9. Comparing the recognition performance using the F1 scores of each emotion and average value, none of the input models with the best performance matched among the drivers. The common results, regardless of the driver, were that “Sad|Fatigued” emotion had the best recognition performance and “Angry|Disgusting” emotion had the worst recognition performance. “Sad|Fatigued” emotion recognition performance was 0.835 and 0.859 and “Excited|Surprised” emotion recognition performance was 0.653 and 0.583 for Drivers A and B, respectively. Both of which are similar performances. However, in the case of “Angry|Disgusting” emotion, recognition performance differed, 0.571 and 0.373 for each driver. Notably, there was very little performance difference between all evaluated models. The difference between the highest and lowest average F1 score was 0.163 and 0.061 for Drivers A and B, respectively. This can be a fail-safe method of the driver emotion recognition model, and each input modal will ensure each other’s redundancy.

## 5. Conclusions

Although real-world datasets for driver emotion recognition are diverse, to overcome the limitation of the lack of consistency in collected data, we proposed a data collection system capable of collecting multimodal datasets during real-world driving. The proposed system was installed in a vehicle and collected the following multimodal data while driving on the real road: videos captured from two viewpoints, audio inside the cabin, driver’s biophysiological data, and vehicle sensor signals via CAN. We designed a self-reportable HMI application to annotate driver emotional states, used as labels for driver emotion recognition. This application allows the driver to select the emotion most similar to their current emotional state among representative emotions. Thus, emotion labels are collected as self-reported emotion labels and no longer inferred by others. In addition, continuous and repeated report requests were made over a long-term period, making the driver’s bias not be reflected in the self-reported emotion label. Since safety is the most important factor in real-world driving, we focused on minimizing drivers’ behavioral and cognitive disturbances in all processes, including sensor selection, flow, and GUI design while designing the data collection system.

According to the results of the data collection experiment in real-world driving, more than 122 h, 4446 km of driving, and 787 GB of data were collected without any accidents. Through statistical analysis of the collected data, the imbalance and report characteristics of self-reported emotion labels were identified, and default and inducible emotions were distinguished. Based on the statistical hypothesis test, the null hypothesis ($H_{0}$) that there is no difference according to the self-reported emotion label for all collected structured data was rejected. The significance of the difference for each driver differed, suggesting the need for personalization of driver emotion recognition. We compared the state-of-the-art face detectors using the collected front images and presented the most suitable face detector and performance evaluation metric for driver face detection. Finally, we conducted a personalized driver emotion recognition study using the collected images and biophysiological and CAN data. The evaluation results of single-modal and multimodal using the above data suggested that multimodal data and personalization are necessary for driver emotion recognition.

Although several case studies were conducted by collecting a large-scale dataset using the proposed system design, enabling safe data collection in real-world driving, the dataset was collected by few drivers over a long period. Because the number of drivers is insufficient to generalize the case studies, these may be treated as particular cases. Based on further collected data, we will continue to study the generalization performance of multimodal personalized driver emotion recognition.

## Figures and Tables

**Table 2:** Detailed information of participated drivers.

	Gender	Age (Year)	Driving Experience (Year)	Experiment Time (h)	Driving Mileage (km)
Driver A	Male	27	more than 15	38	1375
Driver B	Male	32	between 11–15	43	1449
Driver C	Male	26	between 6–10	21	852
Driver D	Male	28	less than 5	20	770

**Table 3:** Statistical hypothesis test results of structured data by self-reported emotion label.

Data		Statistical Hypothesis Test	Post-Hoc Test
		Reject $H_{0}$	Number of Reject $H_{0}$ Pairs (Total Number of Pairs is 6)
Bio-physiological	Skin temperature	Yes	6
	EDA	Yes	5
	PPG	Yes	3
	HR	Yes	4
CAN	Accelerator pedal position	Yes	5
	Brake pedal position	Yes	6
	Steering wheel angle	Yes	6
	Yaw rate	Yes	3
	Longitudinal acceleration	Yes	6
	Lateral acceleration	Yes	5

**Table 4:** Statistical hypothesis test results of structured data by self-reported emotion label according to driver.

Data		Statistical Hypothesis Test				Post-Hoc Test			
		Reject $H_{0}$				Number of Reject $H_{0}$ Pairs (Total Number of Pairs is 6)			
		Driver A	Driver B	Driver C	Driver D	Driver A	Driver B	Driver C	Driver D
Bio-physiological	Skin temperature	Yes	Yes	Yes	Yes	5	6	6	6
	**EDA**	**Yes**	**Yes**	**Yes**	**Yes**	**6**	**6**	**6**	**6**
	PPG	No	Yes	No	Yes	-	1	-	3
	HR	Yes	Yes	Yes	Yes	4	5	5	2
CAN	Accelerator pedal position	Yes	Yes	Yes	Yes	5	6	6	6
	Brake pedal position	Yes	Yes	Yes	Yes	6	5	6	6
	**Steering wheel angle**	**Yes**	**Yes**	**Yes**	**Yes**	**6**	**6**	**6**	**6**
	Yaw rate	Yes	Yes	Yes	Yes	6	6	4	5
	Longitudinal acceleration	Yes	Yes	Yes	Yes	6	5	3	6
	Lateral acceleration	Yes	Yes	Yes	Yes	5	6	6	6

**Table 5:** Driver’s face detection performance comparison of face detectors.

	AP50	AP75	AP95	Speed	GPU
OpenCV	68.4	51.4	0.0	400 FPS	Nvidia GTX 3080
MMOD	83.8	18.1	0.0	260 FPS	Nvidia GTX 3080
MTCNN	81.4	72.0	0.0	4 FPS	Nvidia GTX 3080

**Table 6:** Performance of inducible emotion recognition of Driver A.

	$S_{f}$	$S_{s}$	$S_{b}$	$S_{c}$	$M_{\mathrm{fsb}}$	$M_{\mathrm{fsc}}$	$M_{\mathrm{fbc}}$	$M_{\mathrm{sbc}}$	$M_{\mathrm{fsbc}}$
F1	0.696	**0.698**	0.619	0.355	0.613	0.430	0.469	0.469	0.228
Precision	0.541	0.537	0.478	0.248	0.446	0.280	0.311	0.314	0.231
Recall	0.975	0.998	0.879	0.630	0.982	0.923	0.950	0.927	0.225

**Table 7:** Performance of inducible emotion recognition of Driver B.

	$S_{f}$	$S_{s}$	$S_{b}$	$S_{c}$	$M_{\mathrm{fsb}}$	$M_{\mathrm{fsc}}$	$M_{\mathrm{fbc}}$	$M_{\mathrm{sbc}}$	$M_{\mathrm{fsbc}}$
F1	0.584	0.613	0.593	0.536	0.562	0.646	0.661	**0.667**	0.615
Precision	0.419	0.442	0.475	0.492	0.420	0.539	0.522	0.500	0.468
Recall	0.963	1.000	0.790	0.589	0.852	0.805	0.900	1.000	0.900

**Table 8:** Performance of driver emotion recognition among inducible emotions of Driver A.

		$S_{f}$	$S_{s}$	$S_{b}$	$S_{c}$	$M_{\mathrm{fsb}}$	$M_{\mathrm{fsc}}$	$M_{\mathrm{fbc}}$	$M_{\mathrm{sbc}}$	$M_{\mathrm{fsbc}}$
Average F1		0.496	0.444	0.447	0.561	0.456	0.500	**0.607**	0.557	0.483
Excited	F1	0.359	0.301	0.362	**0.653**	0.344	0.487	0.444	0.465	0.417
|	Precision	0.591	1.000	0.563	0.593	1.000	0.950	0.800	0.909	1.000
Surprised	Recall	0.258	0.177	0.267	0.727	0.208	0.328	0.308	0.313	0.263
Angry	F1	0.293	0.196	0.147	0.263	0.216	0.280	**0.571**	0.400	0.200
|	Precision	0.579	1.000	1.000	0.500	1.000	0.875	0.667	1.000	0.667
Disgusting	Recall	0.196	0.109	0.080	0.179	0.121	0.167	0.500	0.250	0.118
Sad	F1	**0.835**	0.833	0.830	0.768	0.808	0.733	0.807	0.806	0.831
|	Precision	1.000	1.000	1.000	0.977	0.995	1.000	0.926	1.000	1.000
Fatigued	Recall	0.717	0.714	0.710	0.632	0.680	0.578	0.714	0.675	0.711

**Table 9:** Performance of driver emotion recognition among inducible emotions of Driver B.

		$S_{f}$	$S_{s}$	$S_{b}$	$S_{c}$	$M_{\mathrm{fsb}}$	$M_{\mathrm{fsc}}$	$M_{\mathrm{fbc}}$	$M_{\mathrm{sbc}}$	$M_{\mathrm{fsbc}}$
Average F1		0.488	0.472	0.481	0.450	0.491	0.468	0.491	0.501	**0.511**
Excited	F1	0.450	0.403	0.333	0.286	0.511	0.417	0.537	0.511	**0.583**
|	Precision	0.636	1.000	0.452	1.000	1.000	1.000	0.846	0.923	0.539
Surprised	Recall	0.348	0.252	0.264	0.167	0.344	0.263	0.393	0.353	0.636
Angry	F1	0.270	0.270	**0.373**	0.204	0.321	0.194	0.227	0.273	0.233
|	Precision	1.000	1.000	0.452	1.000	0.907	0.429	1.000	1.000	1.000
Disgusting	Recall	0.156	0.156	0.264	0.114	0.195	0.125	0.128	0.158	0.132
Sad	F1	0.744	0.743	0.736	**0.859**	0.641	0.794	0.710	0.719	0.717
|	Precision	1.000	1.000	1.000	1.000	1.000	1.000	1.000	0.958	0.864
Fatigued	Recall	0.593	0.592	0.582	0.753	0.472	0.658	0.550	0.575	0.613

## Abbreviations

The following abbreviations are used in this manuscript:

CAN	Controller area network
HMI	Human–machine interaction
GUI	Graphical user interface
UX	User experience
SAM	Self-assessment manikin
RGB	Red green blue
IR	Infrared
E4	E4 wristband
EDA	Electrodermal activity
PPG	Photoplethysmography
IBI	Interbeat interval
HR	Heart rate
OBD	On-board diagnostics
IoU	Intersection over union
TP	True positive
FP	False positive
PR	Presicion–recall
AP	Average precision
FN	False negative

## Appendix A

The part describes terminologies and variables used in the main text. Table A1 contains details of terminologies and variables.

**Table A1:** Deficition of terminologies and variables used on the main text.

Expression	Definition	Unit
$R_{v}$	Sample rate of the video data	Hz
$R_{a}$	Sample rate of the audio data	Hz
$R_{s}$	Sample rate of the self-reporting	Hz
$R_{c}$	Sample rate of the CAN data	Hz
$I_{r}$	Request time interval of HMI application	s
$I_{rr}$	Re-request time interval of HMI application	s
$I_{s}$	Skip time interval of HMI application	s
*K*	Mileage for completing the train data collection	km
$H_{0}$	Null hypothesis of the statistical hypothesis test	-
$H_{1}$	Alternative hypothesis of the statistical hypothesis test	-
$S_{f}$	Single-modal recognition model of the front image	-
$S_{s}$	Single-modal recognition model of the side image	-
$S_{b}$	Single-modal recognition model of the bio-phyological	-
$S_{c}$	Single-modal recognition model of the CAN	-
*M*	Multimodal recognition model	-
*N*	Total number of representative emotions	-
s	Second	-
bpm	Beats per minute	-
g	Gravitationnal acceleration	$m/s^{2}$
FPS	Frame per second	-
